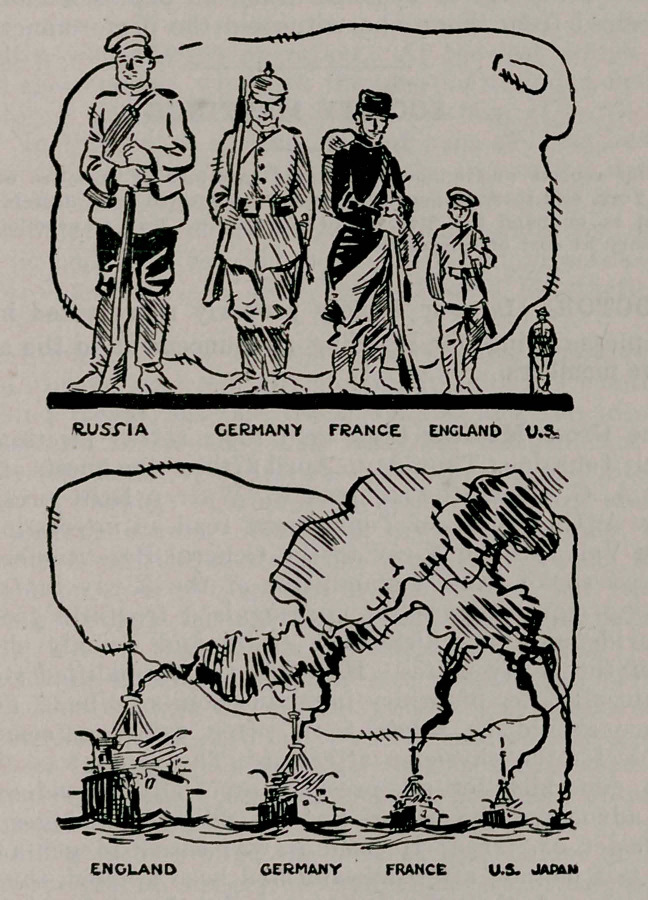# Topics of Public Interest

**Published:** 1916-06

**Authors:** 


### TOPICS OF PUBLIC INTEREST.

**Telephone Merger for Buffalo.** The Chamber of Commerce Committee has reported favorably on the proposed merger of the Federal and Bell companies although the matter will be submitted to telephone users for a vote. The following schedule of rates is proposed:

Individual line business:

| Local Messages to be sent in one year. | Annual Rate. | Additional Local Messages. |
|---|---|---|
| 600 or less | $ 39.00 | 5c each |
| 800 | 48.00 | 5c " |
| 1,000 | 57.00 | 4c " |
| 1,200 | 63.00 | 4c " |
| 1,500 | 72.00 | 4c " |
| 1,800 | 81.00 | 4c " |
| 2,100 | 90.00 | 3c " |
| 2,400 | 97.50 | 3c " |
| 2,700 | 105.00 | 3c " |
| 3,000 | 112.50 | 3c " |
| 3,300 | 120.00 | 3c " |
| *3,600 | 126.00 | 3c " |

*Additional local messages contracted for in advance in lots of 300, two cents each. Messages not contracted for in advance, three cents each.

Extension stations, each, $6.

Auxiliary line and station, $27.

Residence telephones only:

Individual—Flat, $48.

Two-party—Flat, $36.

Four-party—$24 for 600 local messages; additional messages four cents each.

Note—Residence subscribers also have privilege of contracting for residence service at the message rates shown above for business.

While residences and physicians' offices (presumably) are not directly affected, it is a very narrow view to consider only the direct expense. Incoming messages are quite as important and anything that tends to restrict telephone use is an injury to all. The a la carte method of charging is always expensive to the consumer and tends to prevent a free and liberal use of whatever is thus sold. For telephone service, it has the additional disadvantage of distracting the attention of operators from what should be the sole purpose, prompt and reliable connection. If allowed at all, it should be merely to prevent excessive use and while a five cent charge may be considered reasonable to the transient user of a telephone, to cover the cost of maintenance of public booths and some attention both by the company and by the proprietor of the place in which the booth is installed, it is far beyond a proper charge for any regular subscriber. Excessive use automatically tends to correct itself by requiring additional wires and instruments to counteract the serious loss incident to delaying or preventing inbound messages. In England, the prevailing charge for a local telephone call by a transient has been a penny. What Buffalo needs—or any other city—is a local application, if necessary official, of the principle of "The Most Favored Nation." Before submitting to any increase of rates, a thorough investigation should be made of telephone rates throughout the civilized world and an insistance on a minimum of cost and maximum of efficiency. Are our business men so young or so forgetful that they do not realize that the sophistry of the superior economy and efficiency of an unparalleled public service is swept away by the actual facts? Buffalo, except in the early '80's, never had a reasonable rate for telephone service, or a reasonably efficient service, or a service covering anything like the number of persons necessary to convenient communication or even a sufficient support of the company itself. All this has been secured by competition.

---

**The Gasoline Problem.** A letter from an advertising agency to the publisher of a medical journal reads thus: "We are enclosing herewith reprint of an editorial which appeared in the Chicago Herald, April 13. This editorial, we believe, is of great interest to your readers, and we will appreciate your courtesy in having same republished in an early issue of your paper. Such action will also be greatly appreciated by the Standard Oil Co. of———, whose advertising is appearing or shortly will appear in your publication. . . . . . ."

**Attention, The S. P. C. S. !**

The federal trade commission has sent to Congress a preliminary report on the rise in the price of gasoline. It draws no conclusions but presents masses of statistical information. Among the items noted in the press summary are:

Production of crude oil remained virtually stationary; gasoline contents of crude oil decreased; exports of gasoline increased from 188,000,000 gallons in 1913 to 238,500,000 gallons in 1914 and 284,500,000 gallons in 1915; for its 62 per cent of the gasoline produced the Standard Oil Company charged about 1 cent a gallon less than the "independents" charged for their 38 per cent.

The last item ought to move the Society for the Prevention of Cruelty to Statesmen to do something. Consider the hard lot of the member of Congress with a large constituency of automobile owners. Confronted with angry complaints about the "high price of gas" he is deprived of his old familiar explanation.

He cannot dismiss the complaints with the classic vituperation of the "trust"—the "octopus"—for here is the federal trade commission with its cold-blooded price tables. Truly the way of the statesman who deals in oratory meant only "for Buncombe County" grows harder every day.

It will be noted that the figures contradict the report to congress, mentioned in our May issue, which stated that the exports of gasoline had declined from 1914 to 1915, although the differences are not large. According to miles run and consumption per mile, automobiles vary greatly in their use of gasoline and it should be remembered that this use of gasoline is only a part of the total. It would be interesting to have statistics on this point. As an approximate estimate, including heavy commercial vehicles, each motor vehicle uses 1000 gallons a year. The exported gasoline, therefore, would provide for 284,500 automobiles or about 1/8 of the total. The increase since 1913 would provide for about 100,000 or somewhat less than 1/20 of the total. The 1914 figures as well as total yield of crude oil and potential content of gasoline were certainly known to oil men in the summer of 1915, when gasoline was having its maximum use for all purposes and was being sold at 11 cents. The increased exportation for 1915, as compared with 1914—granting that the government statement of a diminished exportation was wrong—corresponded to the consumption of 46,000 automobiles, about 1/50 of the total to be expected for 1916, and to a far less proportion of the total use of gasoline for all purposes. If the production of crude oil remained stationary, that was the fault of the producers. Expert authority holds that there is enough in the ground for 27 years to come, not to mention possible new discoveries and the theory of certain economic geologists that natural gas, and oil are not merely the stationary remains of ancient organic life but that they are being formed by purely inorganic processes. While it must be conceded that the recent production has been low in volatile contents, the recently announced improved method of "cracking," which has been recognized as practicable by the Standard Oil Cos. themselves (note the plural) more than compensates for this. But, concede that oil production remains stationary and that improved processes of cracking merely compensate for the reduction in spontaneous gasoline in crude oil, also that exportation is the determining factor. How does an increase in exportation corresponding to about 2% of the total use of gasoline for automobiles alone, account for a rise in price during the perid of minimum use, amounting to about 150%. The only answer that we can imagine is to be found in the published reports of large dividends and great increase in value of stocks.

We have already called attention to the fact that independent oil companies cannot be relied upon to maintain low prices. We have even spoken of them as assistant standard oil. Still, the admission that 62% of the total production of oil is in the hands of the Standard Cos. is significant.

We also heartily agree with the contention that a congressman cannot "dismiss the complaints with the classic vituperation of the 'trust'". No problem is solved by vituperation but by a calm and sensible consideration of a desired end and of efficient means to reach it. For instance, as a nation, we must get rid of the old idea that all we want is money, expressed by the general policy of a protective tariff on imports and a constitutional objection to restriction or, at least, of taxation, of exports. Various food products are vastly more important than gasoline and, under obvious conditions, it may be more important to retain them than to allow their sale outside the country. Most countries prohibit or at least restrict the exportation of works of art and antiquities. Let it be clearly understood that we do not advocate free trade nor restriction of commerce, nor taurocephalic reduction of prices but all the details concerned must be sensibly considered with a view to attaining the best conditions for the majority of the population, by a reasonably elastic system of control involving import and export taxes or restrictions and any other means necessary.

---

**The Annual Meeting of the Buffalo Assn. for the Relief and Control of Tuberculosis** was held April 28. Moving pictures of the work at the J. N. Adam Memorial Hospital at Perrysburg were shown. The society now owns over 1,500 feet of educational films. Irving S. Underhill is President and Paul E. Batzell, Secretary.

---

**The National Society of Keep Wells** organized by Mrs. Arthur Mac Donald, is progressing in its good work of securing popular conception of hygiene. It is urged that local chapters be formed and suggested that physicians co-operate by giving popular talks. One of the slogans of the society is that a knowledge of the geography of the body is more important than that of the geography of the world.

---

**The Calydor Sanitarium** has been opened at Gravenhurst, Ont., under the management of Dr. C. D. Parfitt. Dr. D. W.

Crombie who has been associated with Dr. Parfitt for three years in sanitarium work, will be resident physician. The sanitarium occupies a new building on Lake Muskoka and is equipped with a bacteriologic and X-ray laboratory. Miss Robina L. Stewart will superintend the nursing, hygiene and dietetics.

---

**U. S. Civil Service Examinations** will be held June 6 to fill the position of Assistant Epidemiologist (male) at $2,000-$2,500; and June 7, for Medical Interne in the Government Hospital for the Insane at $900 and maintenance. We regret that these notices are usually sent out too near the time for due publicity in monthly medical journals.

---

**The Plattsburg Military Training Camp,** is located on Lake Champlain and covers several hundred acres. Last year 1800 business and professional men attended it, 4 from Buffalo. This year the total enrolment on Mch. 31 was 3,088 and the enrolment from Buffalo up to April 20, was 154. It is desired that the total enrolment for this summer be 10,000, of whom 500 should come from western N. Y. Enrolment does not necessitate attendance if subsequently found to be impracticable. The training camps are organized into senior and junior divisions, the former including men from 21 to 45, of high school education or its equivalent, the idea being to limit the camp to men of efficiency and initiative—from which we infer that the idea is to have an available source of officers in case of need. The junior division is for undergraduates or those just graduated from high schools and colleges or graduates still under 21. Senior encampments will be of four weeks each, June 5-July 2, July 12-Aug. 8, Aug. 10-Sept. 6, Sept. 6-Oct. 5. The junior encampment will be July 5-Aug. 8. Expenses beside railroad fare (and strictly personal expenses) will be $30 for seniors and $22.50 for juniors, and a uniform costing about $10. For further information apply to Geo. H. Field, Executive Sec., Military Training Camps Assn., 542 Marine National Bank Bldg., Buffalo. "If you can't go yourself, send someone else."

---

**The Wheel Chair Guild** of Buffalo has acquired as a home for incurables, a large stone house at the corner of Delaware and Kenmore Aves., in Kenmore, just across the city line.

---

**Sun's Rays Focussed on Skin as Substitute for Vision.** L. Zehnder of Berlin, suggests that, as a partial guide for those deprived of sight, the number of blind soldiers being appalling, a lens, with screens, be used to focus light and heat rays on the skin of the chest. He believes that not only would this be of service in orientation but that ideas of the shape and nature sources of reflected light or heat might ultimately be acquired and that, by some arbitrary system, light and heat signals could be used to spell out words.

---

**Over a Hundred Leroy School Children Operated on for Enlarged Tonsils and Adenoids.** Health Officer Graney has secured the services of a Buffalo specialist at a rate of $5 per capita, at least ten pupils to be assembled in the hospital for operation at a time.

---

**German Statistics of Wounds.** In Aug. 1914, 84.8% recovered entirely, 12.2% remained unfit for military service and 3% died. In Aug. 1915, 88.1% were returned to active service and 2.7% died. This shows a wonderfully high efficiency of military surgery. Similar statistics for other countries engaged in the war have also been remarkably good but not so good as these, it is claimed. It is obvious that by no means all military fatalities are instantaneous and we have not been able to ascertain the exact definition of "wounded" as the basis of such statistics as these.

The total German losses to May 1, 1915, are stated at 705,877 killed; 1,781,310 wounded; 334,892 prisoners. (See page 535 May issue.)

---

**Little Damage to The Abbott Laboratories.** A small fire with explosion of gases occurred April 21st on the top floor of one of the buildings of The Abbott Laboratories. Newspaper reports of the extent and character of this accident were grossly exaggerated. The damage was very small, consisting mainly of broken window panes and cracking of temporary partitions. The plant and machinery were injured but slightly, and the entire force went to work the next morning as usual. The Abbott Laboratories have issued a statement positively denying the newspaper reports that this firm is or has been engaged in the manufacture of ammunition or explosives.

---

**Popular Medical Education Through the Lay Press.** As compared with even 25 years ago, the space devoted by the lay press to medical and hygienic matters is very large. Even notes of advances in medical science are usually given prominent positions by most newspapers and are reported quite accurately. Indeed, inaccuracy and exaggeration has seemed to us to occur rather in syndicate matter sent out by quasi-medical organizations for lay instruction. From the standpoint of the medical profession, most of the matter of this sort is trite and not worth repeating but the constant reiteration of hygienic and sanitary dicta must exert a considerable influence on the general health. We note in a syndicate article by Dr. Frank Crane, under the title "If I were king," the following suggestions: Recognize alcohol as a habit-forming-drug and regulate it exactly as heroine and opium . . . to be procured only by physician's prescription. Abolish the Fahrenheit thermometer, the present calendar, substituting one of 13 months, all systems of weights, measures and money not decimal. Abolish criminal courts substituting commissions of psychologists to determine what should be done, not to satisfy public resentment but to cure the defect.

---

**Do You Know That**

1. Walking is the best exercise—and the cheapest?
2. The United States Public Health Service administers typhoid vaccine gratis to Federal employees?
3. A little cough is frequently the warning signal of tuberculosis?
4. Bad teeth and bad tonsils may be the cause of rheumatism?
5. Unpasteurized milk frequently spreads disease?
6. The air-tight dwelling leads but to the grave?
7. Moderation in all things prolongs life?
8. The careless spitter is a public danger?—United States Public Health Service.

1. Just to be contrary, we would reply that the bicycle is better yet because it enables the city resident to get into the country.

2. That we did not know this but that we did know that almost anyone can get free typhoid vaccine somewhere and that we must thresh out the issue of just what forms of medical attendance should be furnished free, as a matter of paternalism, without regard to proper charity to the poor, not in regard to this point alone, but as a general principle in medical ethics and economics.

3. That a little cough is much more often due to something else than tuberculosis and that, in tuberculosis, it occurs a little too late to be considered a warning. It is rather a reminder of neglect.

4. Not that they are strictly causes, though we have occasionally noted cases of rheumatism in which bad teeth and bad tonsils were mobilization points in attacks on the joints and other structures to which the term rheumatism is applicable. We have recently seen a case which had been termed rheumatism and which was diagnosed and proved to be infectious polyarthritis, in which the tonsils and teeth were in exceptionally good condition.

5. Yes but we do not believe that pasteurization will adequately protect against the majority of specific infections which may be carried by milk and we are not yet ready to abandon the old idea of clean, raw milk, protected against gross contamination, and used fresh, before ordinary air bacteria multiply to any dangerous degree.

6. This is our personal opinion (not original) but we know some octogenarians and nonogenarians whose houses are stifling.

7. We used to, but we are getting around to the belief that, excluding practices dangerous on a small scale and actual fatigue and excess, the man who stays till the last dog is hung, prolongs his youth and huskiness.

8. Yes, if there is anything dangerous in his sputum.

---

**Army Surgeons.** The House has adopted an amendment providing for seven medical officers per 1000 troops. Just how large our regular army will be within the next year or two, it is difficult to state but it does not seem that popular sentiment will consent to anything like a permanent solution of the problem on a basis of less than 200,000. This would mean that about 1400 medical officers would be required; an excess of nearly 1000 beyond the present active list. The annual graduation of physicians has fallen to about 3000. According to past experience, about 10% of applicants or would-be applicants for commission, are accepted. The knowledge that the requirements are difficult prevents application in many instances, so that actual statistics of examining boards may show a higher passing proportion. Anything approaching accurate estimates are, of course, impossible but we are inclined to believe that the thousand vacancies in the medical corps which will soon be made, will exhaust the material available, considering the age and exceptional ability demanded by the staff, and the personal predilections of the possible supply. With similar increase in the navy and the numerous other government services, the economic excess of physicians should be remedied to a large degree. It is also worth while considering that a further increase in the proportion of medical work done under the various branches of the government, will occur.

---

**The University of Pennsylvania** by vote of the Trustees, has decided to limit the number of students in the first and the second years of the medical course to 100. This will, with fluctuations due to outside influences, reduce the graduating classes to about 80. In 1915, the graduates numbered 57 but those of Jefferson and the Medico-Chirurgical College which are to be merged with the University of Pennsylvania, were, respectively 145 and 82, a total of 284. While, for economic reasons, we have always strongly favored a reduction in the medical profession, there are certain principles in this country which are opposed to a purely arbitrary limitation of choice of life work and it occurs to us that strictly legal obstacles may exist and that these may possibly apply to raising of requirements when the latter policy is thus formally acknowledged to rest upon a numeric estimate.

---

**The National Security League** of 31 Pine St., N. Y., kindly furnish the accompanying pictorial argument for preparedness.

**The College of Physicians and Surgeons,** Medical Dept. of Columbia University, has opened its doors to women.

---

**The New York State Narcotic Law** (Boylan) had various amendments proposed during the last session of the legislature. These would have still further embarrassed physicians and pharmacists in attempting to follow the spirit of such legislation and we are glad to learn that they have been defeated.

---

**Twilight Sleep** was shown to the public by moving pictures at the Teck Theatre of Buffalo, last month. Men and women were admitted at different performances. The editor was not able to attend the show but doubtless many other physicians did. As many ethical problems have arisen regarding twilight sleep and as some of these are especially connected with this method of demonstration, an expression of opinion is desired from those who witnessed the performance.

## Figures and Tables

**Figure f1:**